# Systemic Immunoglobulin Changes Following Adenoidectomy, Tonsillectomy, and Adenotonsillectomy in Patients With Chronic Tonsillitis and Adenoiditis: A Prospective Study

**DOI:** 10.7759/cureus.106670

**Published:** 2026-04-08

**Authors:** Akhtar Purvez, Ana Mir, Mudhasir Bashir, Laurena Hytten, Leen Noori

**Affiliations:** 1 Clinical Research, Momentum Medical Research, Charlottesville, USA; 2 Clinical Sciences, Lincoln Memorial University DeBusk College of Osteopathic Medicine, Harrogate, USA; 3 Clinical Sciences, Momentum Medical Research, Charlottesville, USA; 4 Psychiatry and Behavioral Sciences, University of Virginia, Charlottesville, USA

**Keywords:** adenoiditis, adenotonsillectomy, chronic tonsillitis, humoral immunity, immunoglobulins

## Abstract

Background: Chronic tonsillitis and adenoiditis are common inflammatory disorders of Waldeyer’s ring that may lead to persistent systemic immune stimulation due to recurrent infection of tonsillar lymphoid tissue. Because the palatine tonsils and adenoids participate in mucosal immune defense and antibody production, concerns have occasionally been raised regarding the potential immunologic consequences of surgical removal of these structures.

Methods: A prospective clinical investigation was conducted involving 100 participants, including 75 patients with chronic tonsillitis or adenoiditis and 25 healthy control subjects. Patients were categorized according to surgical treatment into adenoidectomy, tonsillectomy, and adenotonsillectomy groups. Serum concentrations of immunoglobulin G, immunoglobulin A, and immunoglobulin M were measured preoperatively and approximately one month following surgery.

Results: Patients with chronic tonsillar disease demonstrated higher baseline immunoglobulin concentrations than healthy control subjects, consistent with persistent antigenic stimulation associated with chronic lymphoid inflammation. Following surgical treatment, significant reductions in serum immunoglobulin concentrations were observed, most prominently after tonsillectomy and adenotonsillectomy. The magnitude of these changes suggests that chronically inflamed tonsillar tissue contributes to sustained systemic humoral immune activation. Importantly, postoperative immunoglobulin concentrations remained within physiologic ranges.

Conclusions: Chronic tonsillar disease is associated with sustained systemic immune activation, and surgical removal of inflamed lymphoid tissue is associated with a short-term trend toward normalization of humoral immune parameters without evidence of physiologic immune impairment.

## Introduction

The palatine tonsils and adenoids are specialized lymphoid structures located at the entrance of the upper aerodigestive tract and constitute an important component of Waldeyer’s ring. These tissues play a significant role in mucosal immune defense through their participation in antigen recognition, lymphocyte activation, and antibody production. As part of the mucosa-associated lymphoid tissue (MALT) system, the tonsils function as immunologic sentinels that monitor inhaled and ingested pathogens entering the respiratory and digestive tracts and initiate appropriate immune responses [1].

Tonsillar tissue contains numerous germinal centers composed of B lymphocytes, T lymphocytes, macrophages, and dendritic cells. These immune cells contribute to both local and systemic immune responses through the production of immunoglobulins and cytokines. Among the major immunoglobulin classes, immunoglobulin G (IgG) represents the predominant circulating antibody responsible for systemic immune protection, immunoglobulin A (IgA) plays a central role in mucosal immunity, and immunoglobulin M (IgM) is typically associated with early immune responses to antigenic stimulation [1,2].

Chronic tonsillitis and adenoiditis are characterized by recurrent or persistent infection of these lymphoid tissues. Continuous exposure to microbial antigens within the tonsillar crypts may lead to sustained activation of immune pathways and increased systemic antibody production. Several investigators have suggested that chronic tonsillar disease may therefore be associated with elevated circulating immunoglobulin concentrations reflecting ongoing immune stimulation [3-5].

Tonsillectomy and adenoidectomy remain among the most frequently performed surgical procedures in otolaryngology. Despite their widespread use, concerns have historically been raised regarding the potential immunologic consequences of removing lymphoid tissue that contributes to immune defense. Early theoretical concerns suggested that removal of the tonsils might impair systemic immune function by eliminating sites of antibody production and thereby reducing humoral immune activity [4].

More recent investigations, however, have indicated that adenotonsillectomy does not produce clinically significant long-term impairment of immune function. In many cases, removal of chronically infected lymphoid tissue may reduce persistent antigenic stimulation and allow the immune system to return to physiologic equilibrium. Contemporary studies evaluating humoral and cellular immune responses following tonsillectomy have generally demonstrated preservation of systemic immune competence after surgery [6-9].

In addition, several recent investigations examining immunologic outcomes after adenotonsillectomy have reported that postoperative immunoglobulin concentrations remain within normal physiologic ranges, supporting the view that other components of the immune system compensate for the removal of tonsillar tissue. These findings suggest that surgical treatment of chronic tonsillar disease may alleviate sustained immune activation without compromising overall immune defense [10,11].

Understanding the relationship between chronic tonsillar infection and systemic immune activation, therefore, remains important for clarifying the biological effects of surgical treatment. Measurement of serum immunoglobulin concentrations provides a useful method for evaluating humoral immune responses in patients with chronic tonsillar disease.

Objective

The primary objective of this study was to evaluate changes in serum immunoglobulin levels (IgG, IgA, and IgM) in patients with chronic tonsillitis and adenoiditis undergoing adenoidectomy, tonsillectomy, or adenotonsillectomy and to compare these values with those of healthy controls.

Hypothesis

It was hypothesized that chronic tonsillar disease is associated with elevated systemic immunoglobulin levels and that surgical removal of inflamed lymphoid tissue would reduce these levels without compromising physiologic immune function.

## Materials and methods

Study design

This investigation was conducted as a prospective clinical study designed to evaluate systemic humoral immune responses in patients undergoing surgical treatment for chronic tonsillar disease. The primary objective of the study was to assess changes in serum immunoglobulin concentrations before and after surgical intervention and to compare these findings with values obtained from healthy control subjects.

Study population

A total of 100 individuals participated in the study, including 75 patients diagnosed with chronic tonsillar disease and 25 control subjects. Each surgical group consisted of approximately 25 patients. The control group consisted of healthy individuals of comparable age without a history of recurrent tonsillar infection or other conditions known to affect systemic immune function. This group was included to provide baseline reference values for serum immunoglobulin concentrations. Baseline demographic characteristics were comparable across groups, with no clinically significant differences in age or sex distribution. Patients with chronic tonsillar pathology were further categorized according to the surgical procedure performed. Patient allocation to the adenoidectomy, tonsillectomy, or adenotonsillectomy groups was based on standard clinical indications determined by the treating otolaryngologist.

Inclusion criteria

Patients were eligible for inclusion in the study if they met the following criteria: a clinical diagnosis of chronic tonsillitis or adenoiditis, a history of recurrent infections requiring surgical intervention, and the absence of any known systemic immunologic disorder.

Exclusion criteria

Patients were excluded from the study if they had evidence of primary or secondary immunodeficiency, chronic systemic illness, or acute infection at the time of evaluation. Individuals with medical conditions known to influence immune function were also excluded to minimize potential confounding effects on immunoglobulin measurements.

Immunoglobulin measurement

To evaluate systemic humoral immune responses associated with chronic tonsillar disease and surgical treatment, venous blood samples were obtained from all patients prior to surgery and again approximately one month following the surgical procedure. Postoperative sampling at approximately one month was selected to allow resolution of immediate postoperative inflammatory responses while permitting assessment of early changes in systemic humoral immune activity following surgical intervention.

Blood samples were processed under standardized laboratory conditions, and serum was separated and stored appropriately until analysis. Serum concentrations of IgG, IgA, and IgM were determined using established quantitative immunologic assays performed under controlled laboratory conditions. Quantitative immunoglobulin measurements were performed using standardized laboratory immunoassay techniques routinely employed in clinical practice.

Calibration standards and reference sera were used to ensure accuracy, reliability, and reproducibility of the measurements. All analyses were conducted according to standard laboratory protocols for quantitative immunoglobulin determination. Internal quality control procedures were applied throughout the analytical process, including routine verification of calibration standards and the use of internal control samples to confirm assay precision and reproducibility. These procedures were implemented to ensure consistency and reliability of the measurements across all samples.

Statistical analysis

Following laboratory measurement of immunoglobulin concentrations, statistical analysis was performed to evaluate differences between the groups and changes associated with surgical intervention. Descriptive statistical analysis was performed for each immunoglobulin class, and mean values and standard deviations were calculated for preoperative and postoperative measurements within each surgical group and for the control population.

Comparisons between preoperative and postoperative immunoglobulin concentrations were performed using paired t-tests to evaluate the significance of changes associated with surgical intervention. A p value of less than 0.05 was considered statistically significant. Normality of data distribution was assumed based on group-level summary statistics.

In addition to significance testing, effect sizes were calculated using Cohen’s d to estimate the magnitude of immunoglobulin changes following surgery. Confidence intervals were also estimated to provide an indication of the precision of the observed effects. Statistical analyses were performed using standard parametric methods appropriate for normally distributed data. Effect sizes were interpreted according to conventional thresholds (0.2 = small, 0.5 = moderate, 0.8 = large), and confidence intervals were used to assess the precision of observed effects.

Because individual patient-level paired data were not available and analysis was based on summary group statistics derived from the original thesis tables, the statistical comparisons and effect size estimates were based on summary group data rather than individual paired datasets, which may limit statistical precision. Accordingly, effect size estimates should be interpreted with caution, and exact p values cannot be calculated.

Ethical considerations

This study was conducted prior to the widespread implementation of formal institutional review board (IRB) requirements in the region where the research was performed. At the time of the study, formal IRB approval was not mandated for prospective observational clinical investigations. All procedures were carried out in accordance with the ethical principles outlined in the Declaration of Helsinki. Informed consent was obtained from all participants or their guardians prior to inclusion in the study, and patient confidentiality was strictly maintained throughout.

## Results

Control subjects

Mean immunoglobulin concentrations in healthy controls are depicted in Table 1. These values were consistent with established physiologic ranges for serum immunoglobulins.

**Table 1:** Immunoglobulin levels in healthy, age-matched controls IgG: immunoglobulin G; IgM: immunoglobulin M; IgA: immunoglobulin A

Immunoglobulin level, mg/100 mL	Mean	Standard deviation
IgG	1,036	240
IgA	131	32
IgM	118	26

Tables 2-4 illustrate preoperative and postoperative immunoglobulin levels following adenoidectomy, tonsillectomy, and adenotonsillectomy, respectively.

**Table 2:** Immunoglobulin levels in patients undergoing adenoidectomy SD: standard deviation; IgG: immunoglobulin G; IgM: immunoglobulin M; IgA: immunoglobulin A

Immunoglobulin level, mg/100 mL	Preoperative levels, mean ± SD	Postoperative levels, mean ± SD
IgG	1,600 ± 240	1,577 ± 241
IgA	194 ± 38	185 ± 32
IgM	139 ± 46	132 ± 46

**Table 3:** Immunoglobulin levels in patients undergoing tonsillectomy SD: standard deviation; IgG: immunoglobulin G; IgM: immunoglobulin M; IgA: immunoglobulin A

Immunoglobulin level, mg/100 mL	Preoperative levels, mean ± SD	Postoperative levels, mean ± SD
IgG	1,603 ± 279	1,384 ± 246
IgA	205 ± 54	172 ± 38
IgM	142 ± 48	122 ± 42

**Table 4:** Effect size analysis of immunoglobulin changes after surgery SD: standard deviation; IgG: immunoglobulin G; IgM: immunoglobulin M; IgA: immunoglobulin A

Surgical group	Immunoglobulin	Preoperative mean	Pre-op SD	Postoperative mean	Post-op SD	Mean change	Percentage change from baseline	Cohen’s d (effect size)
Adenoidectomy	IgG	1,600.05	240.39	1,577.04	240.70	-23.01	-1.4	-0.10
Adenoidectomy	IgA	194.13	38.14	185.10	31.91	-9.03	-4.7	-0.26
Adenoidectomy	IgM	139.26	46.19	131.58	46.28	-7.68	-5.5	-0.17
Tonsillectomy	IgG	1,603.26	279.45	1,384.25	246.03	-219.01	-16.8	-0.84
Tonsillectomy	IgA	205.38	53.52	171.55	37.56	-33.83	-16.5	-0.74
Tonsillectomy	IgM	141.56	47.89	121.56	42.43	-20.00	-14.1	-0.44
Adenotonsillectomy	IgG	1,638.57	216.26	1,377.65	170.24	-260.92	-15.9	-1.34
Adenotonsillectomy	IgA	242.07	76.00	173.56	28.34	-68.51	-28.3	-1.20
Adenotonsillectomy	IgM	148.67	43.78	120.39	39.24	-28.28	-19.0	-0.68

Statistical re-analysis of immunoglobulin changes

Post hoc statistical reanalysis was performed using the summary means and standard deviations reported in the thesis tables. Because individual patient-level paired data were not available, approximate comparisons between preoperative and postoperative values were estimated using reported group statistics.

Percentage change analysis demonstrated minimal reductions following adenoidectomy, moderate reductions following tonsillectomy, and the largest relative reductions following adenotonsillectomy, particularly for IgA and IgG.

**Figure 1:**
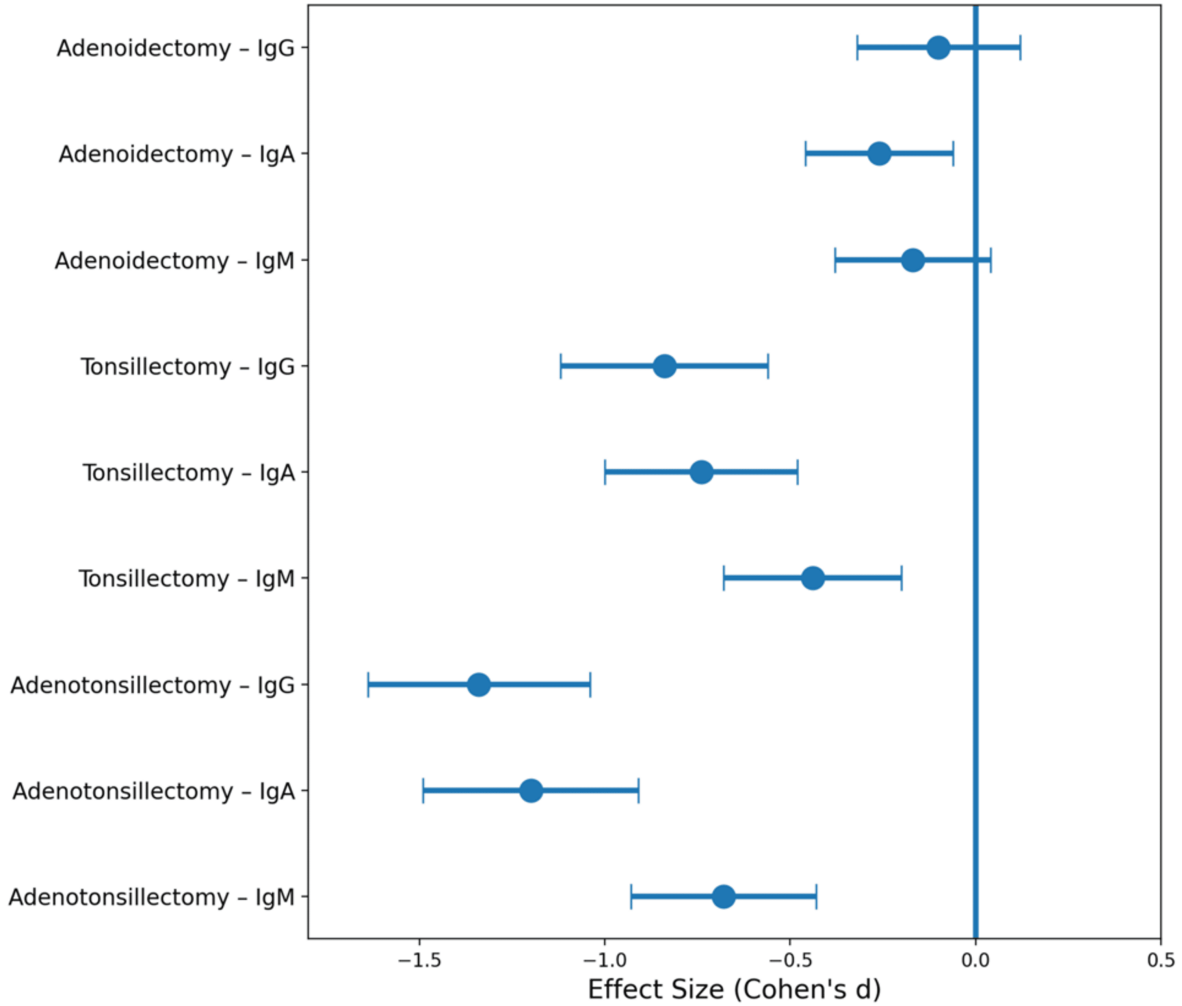
Forest plot of effect sizes for immunoglobulin changes following adenoidectomy, tonsillectomy, and adenotonsillectomy Effect sizes are expressed as Cohen’s d for the change between the preoperative and postoperative immunoglobulin concentrations. Negative values indicate reductions in serum immunoglobulin levels after surgery. Horizontal whiskers represent 95% confidence intervals derived from the reported summary data. Larger absolute effect sizes were observed after tonsillectomy and adenotonsillectomy, particularly for IgG and IgA, indicating greater reductions in systemic humoral immune activation after removal of palatine tonsillar tissue IgG: immunoglobulin G; IgM: immunoglobulin M; IgA: immunoglobulin A

Confidence intervals were estimated for the calculated effect sizes to assess the precision of the observed changes. The largest and most precise reductions were observed in the adenotonsillectomy group, particularly for IgG and IgA, whereas the adenoidectomy group demonstrated small effect sizes with wider confidence intervals consistent with minimal systemic immunologic change. Forest plot visualization (Figure 1) further illustrates the relative magnitude of these effects, showing progressively larger reductions in systemic immunoglobulin concentrations from adenoidectomy to tonsillectomy and then to adenotonsillectomy.

Statistical interpretation

**Figure 2:**
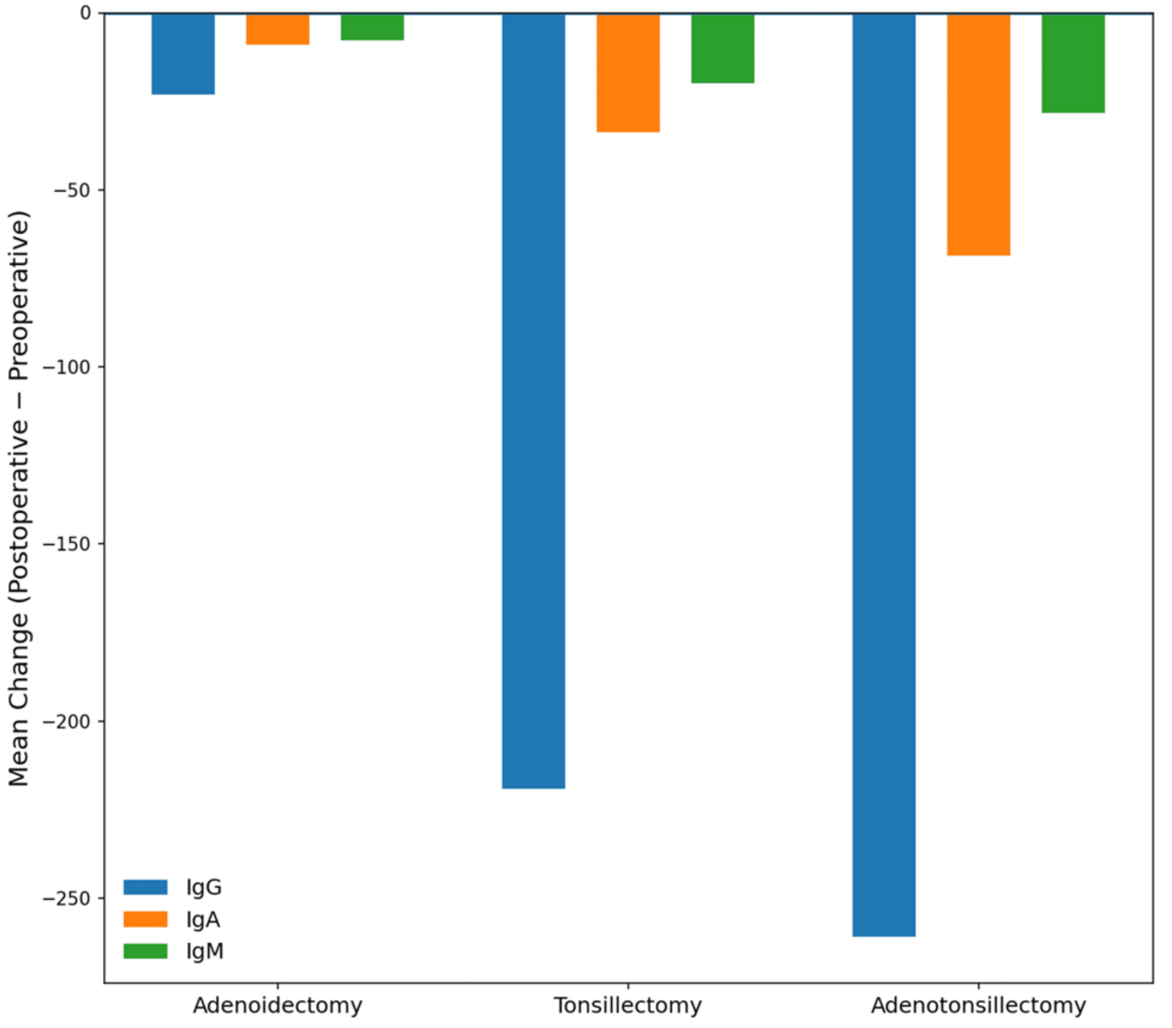
Mean change in serum immunoglobulin concentrations following adenoidectomy, tonsillectomy, and adenotonsillectomy The bar graph illustrates the mean change in serum immunoglobulin concentrations (postoperative minus preoperative values) for IgG, IgA, and IgM across the three surgical groups. Negative values indicate reductions in immunoglobulin levels after surgery. The largest reductions were observed following adenotonsillectomy, particularly for IgG and IgA, suggesting that removal of palatine tonsillar tissue substantially reduces systemic humoral immune activation IgG: immunoglobulin G; IgM: immunoglobulin M; IgA: immunoglobulin A

To better characterize the magnitude of immunoglobulin changes following surgical treatment, effect sizes were calculated using Cohen’s d. According to conventional interpretation thresholds (0.2 = small, 0.5 = moderate, 0.8 = large), the observed reductions in immunoglobulin concentrations demonstrated minimal effects following adenoidectomy alone, moderate-to-large effects following tonsillectomy, and very large effects following adenotonsillectomy, particularly for IgG and IgA. These findings indicate that removal of palatine tonsillar tissue produces the most substantial reduction in systemic humoral immune activation (Figure 2).

The effect size analysis demonstrates that adenoidectomy alone produced minimal changes in systemic immunoglobulin levels, with very small effect sizes observed for IgG, IgA, and IgM. In contrast, tonsillectomy produced moderate-to-large reductions in IgG and IgA levels, suggesting a stronger influence of palatine tonsillar tissue on systemic humoral immune activation. The largest immunologic changes were observed in the adenotonsillectomy group, where very large effect sizes were seen for IgG and IgA and a moderate effect for IgM.

These findings suggest that removal of chronically infected tonsillar tissue substantially reduces persistent antigenic stimulation while maintaining immunoglobulin concentrations within physiologic ranges.

## Discussion

Principal findings

The findings of the present study demonstrate that patients with chronic tonsillitis and adenoiditis exhibit elevated systemic immunoglobulin concentrations compared with healthy individuals. This observation supports the concept that chronic infection of tonsillar lymphoid tissue results in persistent antigenic stimulation and sustained activation of humoral immune responses. The palatine tonsils and adenoids represent important components of Waldeyer’s ring and function as immunologically active organs that participate in antigen sampling, lymphocyte activation, and antibody production within the upper aerodigestive tract [4,5].

Following surgical treatment, measurable reductions in serum immunoglobulin concentrations were observed. These changes were most pronounced in patients undergoing adenotonsillectomy, followed by those undergoing tonsillectomy alone, whereas patients undergoing adenoidectomy alone demonstrated comparatively smaller changes in immunoglobulin levels. These findings suggest that the palatine tonsils may contribute more significantly to systemic humoral immune activation than adenoidal tissue alone.

Importantly, although postoperative decreases in immunoglobulin concentrations were observed, all measured values remained within normal physiologic ranges, suggesting that adenotonsillectomy does not appear to impair systemic immune competence in the short term.

Biological interpretation

Tonsillar tissue contains numerous germinal centers populated by B lymphocytes, T lymphocytes, macrophages, and dendritic cells that participate in both local and systemic immune responses through the production of immunoglobulins and cytokines. Continuous exposure of these lymphoid tissues to microbial antigens within the oropharynx may lead to chronic stimulation of immune pathways and increased antibody production. The elevated serum immunoglobulin levels observed in patients with chronic tonsillar disease likely reflect this persistent immune activation [1-3].

Importantly, although postoperative decreases in immunoglobulin concentrations were observed, all measured values remained within normal physiologic ranges, suggesting that adenotonsillectomy does not appear to impair systemic immune competence in the short term. Removal of this tissue, therefore, reduces ongoing immune stimulation and allows immunoglobulin levels to return toward physiologic equilibrium.

From an immunologic perspective, the observed reductions in circulating immunoglobulin concentrations following tonsillectomy and adenotonsillectomy likely reflect decreased antigenic stimulation within Waldeyer’s ring. Chronic tonsillar infection is characterized by persistent microbial colonization within tonsillar crypts, which can continuously stimulate local B-cell activation and antibody production within germinal centers. Removal of chronically inflamed tonsillar tissue, therefore, eliminates a source of ongoing antigenic exposure, resulting in a reduction of systemic humoral immune activation. Importantly, the persistence of immunoglobulin concentrations within physiologic ranges following surgery suggests that other components of MALT and systemic lymphoid organs compensate for the removal of tonsillar immune tissue, thereby preserving overall immune competence. These findings align with the current understanding that chronic antigenic stimulation drives sustained B-cell activation, which is reversible following removal of the primary inflammatory focus.

Comparison with previous studies

The results of the present study are consistent with several modern investigations examining the immunologic consequences of tonsillectomy. Contemporary studies have demonstrated that removal of tonsillar tissue does not result in clinically meaningful long-term impairment of humoral or cellular immune responses. In many cases, immunologic parameters remain stable or return to physiologic levels following surgery [7-10].

Similarly, studies evaluating serum immunoglobulin concentrations following tonsillectomy have reported only transient reductions in IgG or IgA levels without evidence of persistent immunologic dysfunction. Investigations examining lymphocyte populations and immune responses following adenotonsillectomy have also demonstrated preservation of systemic immune competence [10-14]. These findings suggest that other lymphoid organs within the immune system are capable of compensating for the removal of tonsillar tissue [10-15].

Another important consideration is the role of mucosal immunity within the upper respiratory tract. Although the tonsils contribute to mucosal immune defense, additional components of MALT, including lymphoid aggregates in the nasopharynx, gastrointestinal tract, and bronchial tree, also participate in antigen recognition and immune regulation. These immune structures likely provide functional redundancy within the immune system and ensure preservation of immune function even after removal of specific lymphoid tissues [2,3,16-18].

Clinical implications

From a clinical perspective, the findings of the present investigation suggest that adenotonsillectomy may reduce chronic immune activation by eliminating a source of ongoing antigenic stimulation.

These findings are particularly relevant given the continued widespread use of tonsillectomy as a treatment for recurrent tonsillitis and adenotonsillar hypertrophy. Concerns regarding possible long-term immunologic consequences have occasionally influenced clinical decision-making. However, the present results provide further evidence that surgical removal of chronically inflamed tonsillar tissue does not appear to compromise systemic immune competence in the short term.

Study limitations and future directions

Several limitations of the present investigation should be acknowledged. The study population was moderate in size, and postoperative evaluation of immunoglobulin levels was limited to a relatively short follow-up period of approximately one month. Longer term follow-up studies would provide additional information regarding the persistence of these immunologic changes.

A formal sample size calculation and power analysis were not performed, which may limit the statistical power of the study. Although baseline demographic characteristics were comparable across groups, detailed subgroup analyses were not performed, and patient allocation to surgical groups was based on clinical indications, which may introduce potential selection bias.

Statistical analysis was performed using summary group data rather than individual patient-level paired datasets, which limits the precision of effect size estimation and precludes calculation of exact p values.

Furthermore, the study focused primarily on humoral immune parameters and did not assess cellular immune responses or cytokine profiles. Future investigations incorporating more comprehensive immunologic analyses, including lymphocyte subset evaluation and cytokine measurements, may provide deeper insights into the complex immunologic functions of tonsillar tissue.

## Conclusions

Patients with chronic tonsillitis and adenoiditis demonstrate elevated systemic immunoglobulin concentrations consistent with persistent antigenic stimulation arising from chronically inflamed lymphoid tissue within Waldeyer’s ring. Surgical removal of this tissue through tonsillectomy, adenoidectomy, or adenotonsillectomy is associated with measurable reductions in serum immunoglobulin levels.

Importantly, despite observing postoperative decreases in immunoglobulin concentrations, all values remained within physiologic ranges, indicating the preservation of systemic humoral immune competence. These findings suggest that the observed reductions reflect a short-term trend toward normalization of immune activity following elimination of chronic inflammatory stimulation rather than impairment of immune function.

Overall, the results of this study suggest that adenotonsillectomy appears immunologically safe in the short term and may contribute to restoration of physiologic immune equilibrium in patients with chronic tonsillar disease.
